# Advances in Systemic Lupus Erythematosus Treatment With Monoclonal Antibodies: A Mini-Review

**DOI:** 10.7759/cureus.64090

**Published:** 2024-07-08

**Authors:** Esteban Zavaleta-Monestel, Dina Arrieta-Vega, Carolina Rojas-Chinchilla, Jeimy Campos-Hernández, Jonathan García-Montero, Ricardo Quesada-Villaseñor, Adriana Anchía-Alfaro, Sebastián Arguedas-Chacón

**Affiliations:** 1 Pharmacy, Hospital Clinica Biblica, San Jose, CRI; 2 Rheumatology, Hospital Clínica Bíblica, San José, CRI; 3 Pharmacy, Hospital Clínica Bíblica, San José, CRI; 4 Pharmacy, Universidad de Iberoamérica, San José, CRI; 5 Pharmacy and Clinical Research, Hospital Clínica Bíblica, San José, CRI; 6 Research, Hospital Clinica Biblica, San José, CRI; 7 Research, Hospital Clínica Bíblica, San José, CRI; 8 Pharmacy, Hospital Clinica Biblica, San José, CRI

**Keywords:** pharmacological interventions, monoclonal antibodies (mabs), systemic lupus erythematosus disease, biological therapy, anifrolumab

## Abstract

Systemic lupus erythematosus (SLE) is a chronic autoimmune disease that affects multiple organs and systems. It is characterized by the production of abnormal antibodies that attack healthy cells and tissues. The disease presents a wide range of symptoms and severity, from mild to severe. Diagnosis can be complex, but the classification criteria of the American College of Rheumatology (ACR) help to facilitate it. Incidence and prevalence vary considerably worldwide, mainly affecting adult women between the third and fourth decades of life, although it can also occur in childhood. The prognosis of SLE has improved over time, but there is still a risk of irreversible organ damage. Treatment is individualized for each patient and is based on immunosuppression and the use of corticosteroids. Biological therapies, such as monoclonal antibodies, have emerged as a more specific alternative. Methotrexate, antimalarials, glucocorticoids, immunosuppressants, and monoclonal antibodies are some of the medications used to treat SLE. New therapeutic strategies are currently being developed, such as targeted therapies, immunomodulators, and biological agents. Treatment adherence, monitoring, and regular follow-up are important aspects of SLE management. This article aims to describe the characteristics of the new monoclonal antibody therapies that exist for the management of SLE.

## Introduction and background

Systemic lupus erythematosus (SLE) is a chronic autoimmune disease that has captured the medical community's attention for centuries. Since its first description over 500 years ago, the understanding of SLE has significantly evolved, reflecting increased knowledge and improved individualization of the disease [1]. SLE is characterized by the formation of abnormal antibodies that attack the body's healthy cells and tissues. This action triggers an uncontrolled inflammatory response, primarily in the connective tissue, affecting various organs and systems. Consequently, if not properly treated, the disease can progress, causing irreversible damage to organs, such as the kidneys, heart, lungs, and central nervous system, and may even lead to death [2-4].

The heterogeneous nature of SLE, with a wide range of symptoms and presentations, has made accurate diagnosis difficult. The absence of a pathognomonic profile or specific laboratory tests represents a significant challenge for physicians treating SLE patients. Consequently, predicting the course of the disease and preventing irreversible damage to organs and systems are difficult objectives to achieve [5].

In response to these challenges, experts from the American Rheumatism Association (now the American College of Rheumatology) developed more precise classification criteria for SLE. These criteria not only facilitate diagnosis but also allow for uniform comparison among different patients. The improvement in diagnostic accuracy and classification of SLE enables a better understanding of the disease, leading to the implementation of more personalized and effective therapeutic strategies [1,6].

SLE exhibits a wide range of clinical manifestations among patients. Some cases only experience mild joint and skin involvement, while others suffer from severe, life-threatening complications, such as renal, hematologic, or central nervous system involvement [7,8]. The primary symptoms of SLE include recurrent inflammations, skin infections, cardiovascular problems, kidney diseases, anemia, and sepsis. Among these, lupus nephritis is considered one of the most severe complications [9-13].

Photosensitivity is a common symptom of lupus, affecting a large percentage of patients. This sensitivity causes skin lesions, such as red spots and bumps, to appear on any part of the body, especially in areas exposed to the sun. Cutaneous vasculitis, present in about 20% of cases, is characterized by inflammation of the small blood vessels in the skin, manifesting as reddish or purple rashes, primarily on the legs, bony prominences, and sometimes on the palms of the hands and the soles of the feet [14].

The antibodies produced by the immune system mistakenly target components of the cell nucleus, especially double-stranded DNA. This action generates immune complexes that circulate in the blood and deposit in various organs, causing tissue damage. Among the most severe consequences is persistent proteinuria, which can lead to chronic renal failure and, in some cases, death [15].

The clinical presentation of SLE is highly heterogeneous, with patients experiencing only some of the possible clinical features [16]. In recent years, it has been observed that SLE primarily affects adult women between the third and fourth decades of life, although it can also occur in childhood, with increased severity in the latter case. The disease may occur due to race, gender, or age, but it has a higher incidence and prevalence in Afro-Caribbean, Afro-American, and Asian individuals [2,3].

The incidence and prevalence of SLE show significant variation worldwide. Various factors influence these disparities, such as access to healthcare, environmental exposures, socioeconomic status, genetic predisposition, and the heterogeneity of the disease [16]. Overall, there is an increase in the frequency of SLE, mainly due to the early detection of mild cases. Incidence and prevalence rates vary considerably by region. In the United States, an annual incidence of 5.1 per 100,000 population is estimated. In Western Europe, the annual incidence ranges from 2.2 to 4.7 per 100,000 population, while in the UK, the annual incidence can reach 22 per 100,000 population in the Afro-Caribbean population [1].

The complexity of SLE lies in the difficulty of anticipating its development and preventing irreversible damage to organs, as mentioned earlier, which impacts the patient's prognosis. While survival rates have improved, individuals with SLE still have a standardized mortality rate 4.6 times higher than the general population. Prolonged survival entails greater exposure to medications to control disease activity over an extended period. Both disease activity and pharmacological treatment can predispose to permanent damage [5].

While survival rates in SLE have improved, treatment is still far from perfect. Total disease control is often not achieved due to the variety and severity of clinical manifestations, the possibility of flares, and the toxicity associated with long-term use of oral corticosteroids (OCS) and immunosuppressants. SLE harms all aspects of health, including physical and mental health, vitality, pain, and social and emotional functioning [12].

In the treatment of lupus erythematosus, immunosuppression is a cornerstone, but it also poses a risk to the patient. In this context, biological therapies have emerged as a more specific and safer alternative [17]. Monoclonal antibodies are a type of biological therapy obtained from the fusion in culture of a B lymphocyte and a myeloma cell. These antibodies can recognize and bind to specific targets of the immune system, allowing for the modulation of its activity and the control of the disease in a more specific manner. The advantages of monoclonal antibodies in the treatment of lupus erythematosus include greater efficacy, lower toxicity, and improved quality of life for patients. However, further research is still needed to determine the long-term effectiveness of these therapies and optimize their use in different patient populations [18].

## Review

Fundamental principles for managing SLE

In the management of SLE, there are fundamental principles that guide the approach toward the disease and patient well-being. These principles are essential pillars for effective and personalized treatment.

Individualized Management

SLE is a complex disease that requires a comprehensive and multidisciplinary approach for effective management. While rheumatologists are the primary specialists, the active involvement of other medical disciplines such as dermatologists, nephrologists, and hematologists, among others, is essential to address the various manifestations of the disease and ensure patient well-being [19]. This multidisciplinary approach should be centered on the needs and preferences of each patient, considering their lifestyle, comorbidities, and psychosocial aspects. Patient education is crucial for the success of treatment so that the patient can understand the disease and the importance of adhering to the treatment [20].

Clinical Evaluation

The frequency of medical reviews for patients with lupus varies and depends on the individual characteristics of each case. This can range from a few days, as is the case for patients with lupus nephritis, to six months. The decision on the frequency of visits is made by the treating physician, who relies on various criteria, including disease activity (assessed using instruments such as SELENA-SLEDAI or SLEDAI-2K) and the presence of irreversible damage. Periodic assessment of damage is crucial, as accumulation has significant prognostic value for the patient [21,22].

Non-pharmacological Interventions

Avoiding sun exposure is particularly important due to the characteristic photosensitivity of the disease. Similarly, the importance of quitting smoking should be emphasized [23], as this habit is not only harmful to overall health but can also interfere with the effectiveness of some medications used to treat lupus, such as antimalarials and biologicals like belimumab [24].

Pharmacological Interventions

Pharmacological treatment should be personalized for each patient, considering the variability in disease presentation and individual response to medications. This individualization is crucial due to the phenotypic heterogeneity of lupus, the variable severity of organ involvement, and the differential susceptibility to drugs based on patient characteristics [25]. In patients with mild symptoms, monotherapy with hydroxychloroquine may be sufficient, while in patients with severe disease involving organs, more potent immunosuppressive medications such as high-dose glucocorticoids and cyclophosphamide are required [25]. When selecting therapy, it is essential to consider factors such as race and ethnicity, as Black patients with lupus nephritis tend to respond better to mycophenolate than to cyclophosphamide. In addition, socioeconomic determinants and access to different medications should be considered [26].

Early Diagnosis of SLE

Late diagnosis of SLE remains a common issue, underscoring the urgency of early diagnosis and close monitoring, especially during the initial stages of the disease. This allows for the timely identification and treatment of organ damage, improves quality of life, and reduces the risk of serious complications [27]. Establishing personalized therapeutic goals and promoting patient adherence to treatment are fundamental pillars for success. Non-adherence to treatment is a significant cause of therapeutic failure, so providing education, support, and strategies to the patient is crucial to ensure the long-term effectiveness of treatment [28]. A proactive approach that combines early diagnosis, vigilant monitoring, establishment of personalized therapeutic goals, and promotion of treatment adherence is essential to optimize outcomes in patients with SLE [29].

Primary therapeutic approaches for SLE

Methotrexate

This compound functions as a folic acid antagonist, also known as pteroylglutamic acid. Its primary mechanism of action involves a reduction in intracellular tetrahydrofolate (THF) concentrations. This effect is achieved through the inhibition of two key enzymes: dihydrofolate reductase (DHFR) and thymidylate synthase [30]. As a result, there is a disruption in the S phase of the cell cycle, which explains many of its side effects. Nevertheless, this mechanism also produces beneficial therapeutic effects, such as antiproliferative, anti-inflammatory, and immunoregulatory actions, which together contribute to its efficacy in the treatment of various diseases [31]. Several studies have confirmed the effectiveness of methotrexate in the treatment of SLE. This autoimmune disease primarily affects the joints, causing arthritis that can lead to deformities. Methotrexate has proven to be beneficial in 80% of patients with SLE who tend to develop arthritis. Its use helps control the skin manifestations of the disease, thereby reducing the need for corticosteroids [32].

Antimalarial Medications

Antimalarial medications, such as hydroxychloroquine and chloroquine, are useful in the treatment of patients with SLE who do not have severe organ involvement. These therapeutic options are primarily employed to control the articular, cutaneous, and systemic manifestations of the disease [33]. Hydroxychloroquine, with its immunosuppressive and anti-inflammatory properties, is positioned as a first-line medication in the treatment of SLE. Its efficacy in controlling disease activity, along with generally acceptable side effects, makes it an attractive option for patients. In addition, its ability to improve skin damage, photosensitivity, and mild arthritis makes it a valuable tool for the comprehensive management of SLE [34]. In the case of chloroquine, it has also proven effective in controlling SLE activity, particularly in treating skin damage. When combined with methotrexate, it can help reduce the need for corticosteroids, thereby decreasing the likelihood of adverse effects. Both hydroxychloroquine and chloroquine have demonstrated the ability to increase the long-term survival of patients with SLE and prevent disease flare-ups [35].

*Glucocorticoids * 

Glucocorticoids, such as prednisone, are fundamental in the treatment of SLE, especially in cases of moderate to severe activity. Their effectiveness in inducing rapid disease remission has made them the standard therapy, with tapering regimens not yet well defined. However, it is important to highlight that they are also the primary cause of SLE-related toxicity [36]. Glucocorticoids have their effect, which is based on two important mechanisms of action as the main genomic pathway where the glucocorticoid binds to its specific receptor, the cytoplasm glucocorticoid receptor (cGR), within the cell. This receptor-ligand complex moves to the cell nucleus. It acts as a regulator of gene expression where it inhibits the production of cytokines and other inflammatory proteins, which generates its anti-inflammatory effect and activates the transcription of genes involved in the body’s metabolic response. It is important to note that if glucocorticoid concentrations at the nuclear level increase, side effects may occur. [37]

The second mechanism of action is the non-genomic pathway where glucocorticoid acts directly on inflammatory and immune cells and modulates the activity of cell membranes and intracellular signaling, producing rapid and independent effects of gene transcription. Although glucocorticoids are an important tool for managing SLE, their use must be carefully monitored by a specialist due to their potential to cause short- and long-term side effects [38,39]. High and low doses of glucocorticoids, as shown in Table 1, and the activation of the non-genomic pathway begins at doses greater than 100 mg per day of prednisone or its equivalent [37].

Immunosuppressive Medications

Immunosuppressive medications are a fundamental tool for controlling SLE. These drugs act on the cells of the immune system, responsible for the inflammation and tissue damage characteristic of the disease. Among the most used immunosuppressants in SLE are alkylating agents such as cyclophosphamide, which could destroy proliferating immune cells; inosine monophosphate dehydrogenase (IMPDH) inhibitors such as mycophenolate mofetil and mycophenolic acid, which block the synthesis of nucleotides essential for cell division; and selective inhibitors of purine or pyrimidine synthesis such as azathioprine, which interfere with the production of basic components of DNA necessary for cell replication [40].

The combination of different immunosuppressants with distinct mechanisms of action allows for a synergistic effect and greater efficacy in controlling SLE. In this way, inflammation can be reduced, tissue damage prevented, and the quality of life of patients improved [40].

The European League Against Rheumatism (EULAR) recommends reserving treatment with immunosuppressants for patients with SLE who do not respond to treatment with antimalarials or glucocorticoids. This includes patients who do not experience an improvement in disease activity with initial therapy or who cannot reduce the dose of glucocorticoids to a safe level for chronic treatment. Therefore, their use should be carefully considered and reserved for those patients who truly need them [41].

Monoclonal antibodies for the treatment of SLE

Anifrolumab

Anifrolumab is a monoclonal antibody that has been approved by the FDA for the treatment of moderate to severe SLE in patients receiving standard therapy. Its mechanism of action is based on blocking the receptor of type I interferon (IFN), a cytokine that is elevated in many patients with SLE [42]. Type I interferon plays a crucial role in the body's antiviral response, but it can also contribute to inflammation and tissue damage in SLE. By blocking the activity of type I IFN, anifrolumab can help control inflammation and improve disease symptoms. Clinical studies have shown that anifrolumab is effective in reducing lupus activity, the frequency of flares, and the need for corticosteroids [43-45].

A comparative study between anifrolumab and belimumab in patients with SLE revealed that those who received anifrolumab were more than twice as likely to experience a reduction in disease activity [46]. Several additional studies have corroborated the efficacy of anifrolumab in the treatment of SLE, although adverse events such as upper respiratory tract infections, nasopharyngitis, bronchitis, and herpes zoster have also been documented [47].

Multiple investigations have confirmed that treatment with anifrolumab offers the possibility of reducing or eliminating the need for glucocorticoids in patients with SLE. Approximately 90% of patients receiving anifrolumab experience a reduction in disease activity without the need to initiate or continue glucocorticoid therapy [48]. The gradual and sustained reduction of glucocorticoids leads to clinical benefits, and anifrolumab has proven effective in this regard. A study showed that 51% of patients who received anifrolumab achieved a gradual reduction in glucocorticoid dose and a decrease in overall disease activity, compared to 32% of patients who received a placebo. These results confirm the potential of anifrolumab to improve the quality of life of patients with SLE [49].

Belimumab

Belimumab is a monoclonal antibody that targets the immune system. However, instead of directly targeting B cells, it targets B-cell activating factor (BAFF). This approach allows for more precise action and fewer side effects. Belimumab has been approved by the FDA as adjunctive therapy for patients with active SLE who do not respond to conventional treatment [39]. It is used in adults with active skin or joint disease, and it is one of the approved therapies for SLE including lupus nephritis [36,45,50]. Its main action involves binding to the soluble BLyS receptor, preventing the survival of B cells and the production of immunoglobulins by plasma cells. This effect reduces inflammation and lupus symptoms. It is important to note that belimumab may take three to six months to reach its maximum effectiveness. Therefore, in patients with severe diseases, it is often combined with other medications that act more rapidly, such as glucocorticoids or immunosuppressants [43,51].

In a phase 3 clinical trial involving patients with lupus nephritis, the efficacy of belimumab was evaluated compared to placebo, both administered alongside standard therapy (cyclophosphamide/azathioprine or mycophenolate mofetil). The results indicated that belimumab was significantly more effective in improving renal response in patients with proliferative lupus nephritis. In addition, a significant reduction in the risk of renal events or death and lupus nephritis flares was observed in the overall population receiving belimumab. Compared to standard therapy, this drug also reduced the risk of a sustained 30% to 40% decrease in the estimated glomerular filtration rate [50-52].

An analysis of patients from Hong Kong, China, South Korea, and Taiwan showed that belimumab reduces the risk of death or renal events compared to placebo. In addition, a study based on a US database found that belimumab was associated with a lower risk of severe infection than oral immunosuppressants. This finding makes it an option to consider in the risk/benefit assessment for the treatment of SLE [53].

Rituximab

Rituximab is a monoclonal antibody designed to target specific diseases. Its action is based on precise binding to B cells carrying the CD20 marker. This interaction triggers a series of events that culminate in programmed cell death, or apoptosis, of the affected B cells. In addition, rituximab's action induces the destruction of these cells through other mechanisms of the immune system, such as complement-dependent cytotoxicity, antibody-dependent cellular cytotoxicity, and phagocytosis. By eliminating the B cells responsible for producing pathogenic antibodies, rituximab interrupts their differentiation into plasma cells, which in turn reduces the quantity of these harmful antibodies and helps control the disease [45].

The EXPLORER trial evaluated the safety and tolerability of rituximab in patients with moderate to severe SLE. The results indicated that rituximab showed a safety profile similar to placebo, with no significant differences in the rate of serious adverse events [54]. In subgroups of African American and Hispanic patients, rituximab showed a beneficial effect [55]. The trial demonstrated a trend toward improvement in disease activity for patients with moderate to severe SLE treated with rituximab. However, this improvement did not reach statistical significance, indicating that rituximab was not definitively superior to placebo in the primary clinical outcomes. Nonetheless, a post hoc analysis suggested a potential benefit of rituximab treatment, with a possible reduction in the incidence of severe SLE flares. In conclusion, while the EXPLORER trial did not establish rituximab's superiority for disease control, it provided valuable insights into the safety and tolerability of rituximab in this patient population [56].

Secukinumab

Secukinumab is a monoclonal antibody of high specificity and a promising therapeutic optic for the management of SLE [57]. Its mechanism of action is based on the neutralization of interleukin-17 (IL-17), a pro-inflammatory cytokine that plays a crucial role in the pathogenesis of this autoimmune disease. IL-17-producing T helper 17 (Th17) cells become the primary target of secukinumab [58,59]. These abnormal immune cells infiltrate tissues and organs, releasing pro-inflammatory cytokines that attract more immune cells and amplify the inflammatory response. This causes tissue damage and causes the debilitating symptoms of SLE [58].

Secukinumab binds to IL-17 with high affinity, blocking its interaction with the IL-17R receptor. This binding neutralizes the inflammatory signal, calming the cytokine storm and allowing the immune system to restore balance. Studies have shown that secukinumab can significantly reduce IL-17 levels in patients with SLE, resulting in improved symptoms and higher quality of life. Red skin and flares decrease, fatigue and joint pain are relieved, and internal inflammations are controlled [60].

B-cell targeting strategies for improved lupus management

B cells play a crucial role in the immune system, producing antibodies that fight infections. However, in SLE, B cells lose their ability to distinguish between self and non-self-cells, generating autoantibodies that attack healthy tissues in the body [57]. BAFF plays a crucial role in the survival, maturation, and function of B cells, which are involved in the pathogenesis of SLE. It is important to note that B cells are involved in the pathogenesis of SLE through both antibody-dependent and antibody-independent mechanisms [61].

Antibodies produced by B cells trigger an inflammatory response in SLE. Belimumab, a monoclonal antibody that specifically binds to soluble BAFF, became the first and only FDA-approved BADD inhibitor for the treatment of SLE. Following the success of belimumab, other molecules emerged to inhibit BAFF and its impact on SLE. These molecules had different mechanisms of action and targeted different forms of BAFF, such as blisibimod, tabalumab, atacicept, bortezomib, ofatumumab, ocrelizumab, daratumumab, epratuzumab, fenebrutinib, obexelimab, and rituximab [62].

Table 1 details the doses used in both the conventional treatment of SLE and in the administration of monoclonal antibodies for its management [41,57,63-69].

**Table 1:** Dosage of medications used in SLE * Secukinumab dose approved by the FDA for use of psoriasis in plaque. Abbreviations: IV: intravenous, SLE: systemic lupus erythematosus

Treatment	Doses
Immunomodulators	
Hydroxychloroquine:	5 mg/kg of body weight per day
Glucocorticoids	
High dose, short duration, IV	250 mg to 1 g IV per day for three to five days
Moderate to high dose	>7.5 mg up to 1 mg/kg/day
Low dose	<7.5 mg/day
Non-Biologic immunosuppressants	
Azathioprine:	≤2 mg/kg/day
Cyclophosphamide:	500 mg IV every two weeks for six doses (preferred); 500-1000 mg/m^2^ every month for six doses (alternative)
Methotrexate	≤25 mg/week in combination with folic acid
Mycophenolate mofetil/mycophenolic acid:	Mycophenolate mofetil: ≤3000 mg/day divided into two doses mycophenolic acid: ≤2160 mg/day divided into two doses
Cyclosporine	3.5 mg/kg/day divided into two doses
Tacrolimus:	Typical range: 3-5 mg/day divided into two doses
Biologic agents	
Anifrolumab	300 mg IV every four weeks
Belimumab	10 mg/kg IV every four weeks or 200 mg subcutaneously weekly
Rituximab	1 g IV twice separated by two weeks or 375 mg/m2 IV weekly for four doses
*Secukinumab	300 mg subcutaneously every week for the first five weeks and then 300 mg subcutaneously every four weeks

## Conclusions

SLE, being a chronic autoimmune disease, can affect various organs and systems of the body, generating a wide range of symptoms and severities. However, ACR criteria help facilitate the diagnosis and identification of the disease. SLE can occur at any age, with a predominance in recent years among women in the third and fourth decades of life. It manifests as recurrent inflammations, skin infections, cardiovascular problems, kidney diseases, anemia, and sepsis, with lupus nephritis being one of the most severe complications of this disease. The prognosis of SLE has improved over time due to the availability of therapies, but there is still a risk of irreversible organ damage in these patients. Treatment is based on immunosuppression and the use of corticosteroids, aiming to individualize treatment for each patient. Currently, new therapies for SLE are being developed, such as anifrolumab and belimumab, which, as two approved monoclonal antibodies for the treatment of SLE, have proven effective in reducing both the severity and frequency of the disease. Early detection and treatment are crucial for improving the quality of life of these patients.
